# Plants have two minds as we do

**DOI:** 10.1080/15592324.2025.2474895

**Published:** 2025-03-11

**Authors:** Tomonori Kawano, Yoshiaki Ushifusa, Stefano Mancuso, Frantisek Baluška, Lucia Sylvain-Bonfanti, Delphine Arbelet-Bonnin, François Bouteau

**Affiliations:** a International Photosynthesis Industrialization Research Center, Faculty and Graduate School of Environmental Engineering, The University of Kitakyushu, Kitakyushu, Japan; b Paris Interdisciplinary Energy Research Institute (PIERI), Paris, France; c University of Florence LINV Kitakyushu Research Center (LINV@Kitakyushu), Kitakyushu, Japan; d Advanced Photonics Technology Development Group, RIKEN Center for Advanced Photonics, Saitama, Japan; e Faculty of Economics and Business Administration, The University of Kitakyushu, Kitakyushu, Japan; f Université Paris-Cité, laboratoire dynamiques sociales et recomposition des espaces (LADYSS UMR 7533), Paris, France; g LINV-DiSPAA, Department of Agri-Food and Environmental Science, University of Florence, Sesto Fiorentino, FI, Italy; h Institute of Cellular and Molecular Botany, University of Bonn, Bonn, Germany; i Laboratoire Interdisciplinaire Des Énergies de Demain, Université de Paris-Cité, Paris, France

**Keywords:** Plant behavior, prospect theory, probability, subitizing, two minds, weighting function

## Abstract

This discussion paper carefully analyzes the cognition-related theories proposed for behavioral economics, to expand the concepts from human behaviors to those of plants. Behavioral economists analyze the roles of the intuitive sense and the rational thoughts affecting the human behavior, by employing the psychology-based models such as Two Minds theory (TMT) highlighting intuitive rapid thoughts (System 1) and rational slower thoughts (System 2) and Prospect theory (PT) with probability (*p*)-weighting functions explaining the human tendencies to overrate the low *p* events and to underrate the high *p* events. There are similarities between non-consciously processed System 1 (of TMT) and overweighing of low-*p* events (as in PT) and also, between the consciously processed System 2 (of TMT) and underrating of high-*p* events (as in PT). While most known *p*-weighting mathematical models employed single functions, we propose a pair of Hill-type functions reflecting the collective behaviors of two types of automata corresponding to intuition (System 1) and rationality (System 2), as a metaphor to the natural light processing in layered plant leaves. Then, the model was applied to two different TMT/PT-related behaviors, namely, preference reversal and habituation. Furthermore, we highlight the behaviors of plants through the above conceptual frameworks implying that plants behave as if they have Two Minds. Lastly, the possible evolutionary origins of the nature of Two Minds are discussed.

## Introduction

A pioneering researcher in behavioral economics, D. Kahneman (1934–2024; known for the 2022 Nobel Memorial Prize in Economic Sciences) and his colleagues have developed a key theory for human behaviors known as Two Minds theory (TMT) explaining the rapid and slow mental functions or thoughts^1,2^ behind the cognitions^3^ and morals.^4^ In TMT, the rapid thought designated as System 1 is considered to be non-consciously processed and responsible for intuition and indignation, and the thought called System 2 represents the conscious, deliberate, and rational phase of mental functions.

The concept of Two Minds was developed after the series of earlier works known as Prospect theory (PT) which were also documented by Kahneman’s team.^5,6^ PT handles two distinct modes of cognitions, namely, the rapid and non-conscious cognitive mode responding to low-probability (*p*) events often overrating the values, and the slow but rational mode of cognition responding to high-*p* events often underrating the values. Due to the continuity of the series of studies by the same group, similarity could be found between System 1 (of TMT) and the cognition of low-*p* events (in PT), and between System 2 (of TMT) and the cognition of high-*p* events (in PT).

In order to discuss if plants have “Two Minds” as we humans do, this discussion paper carefully analyzes the psychology-assisted cognition-related theories widely accepted in the area of behavioral economics, and attempted to expand the corpus of experimentally testifiable phenomena to cover the behaviors of plants.

### Sense of number and discrete mathematics

Discrete mathematics is the basis for a wide range of mathematical disciplines such as the studies on enumeration, block designs, the combinatorics of partially ordered sets, algebraic combinatorics, discrete geometry, and matrices, by covering the theories of graph, coding, discrete probability, extremal set, and matroid.^7^ Some researchers such as K. D. Joshi^8^ viewed that discrete mathematics began at least as early as humans or perhaps some other animals learned to count. It is obvious that, regardless of personal educational history, human individuals can instantly recognize the basal numeric nature of the objects or phenomena that advent before eyes, e.g., if the objects are composed of 2 or 3 elements.

Cognitive scientists have revealed that humans at their very infancy (even neonates) can distinguish the difference between the groups made up of two and three,^9^ or between three and four^10^ at third to fourth days after the birth. At the fourth month after the birth, human infants developed enough to understand minimal addition and subtraction such as 1 + 1 = 2, and 2 − 1 = 1,^11^ and this developmental stage is soon followed by the acquisition of the sense of arithmetic operations with larger numbers such as 2 + 1 = 3 and 3 − 1 = 2.^12^

### Subitizing for small numbers

This ability based on the primary sense of number is named as “subitizing” according to a pioneering cognition researcher P. E. L. Kaufmann^13^ and related works.^14,15^ The subitizing capability is known to be shared among primates such as humans and chimpanzees.^16,17^ In addition to primate species, there are plenty of evidence that the subitizing capability could be also found in wild non-primate mammals including lions,^18^ and also in a variety of animals such as birds,^19^ fishes,^20^ and even insects.^21^ It is also tempting to speculate that plants and microorganisms can count and compute as in the cases of nerve plants such as Venus flytrap (*Dionaea muscipula*) counting the number of mechanical stimuli,^22^ unicellular organisms such as green paramecia^23–25^ and aquatic bacteria^23,26^ possibly counting the numbers of self- and nonself-cells by behaving as living (cellular) automata in order to make decisions for actions or survival. It is worthy to share the knowledge on the biological computation naturally performed with a slime mold.^27,28^ Furthermore, sub-cellular components in biological systems such as deoxyribonucleic acid (DNA) and proteins can be used as molecular automata for semi-natural computing operations.^7,29,30^

As found in the above cases, the primitive numeric capability behind the subitizing should be free from education and the ability to ‘memorize’ the tables for additions and multiplications, therefore, the numerical ability should be dissected to the subitizing for a handful of small numbers and the rational calculations with greater numbers. In humans, the primitive numerical capability is now known to be rooted in the function of the angular gyrus, a region of the brain, occupying the posterior part of the inferior parietal lobule, apparently distinct from the memorizing function of the basal ganglia, a group of subcortical nuclei found in the brains.^14,19^

By combination of multiple functions of brains, simple arithmetic operations required for daily life could be performed through subitizing capability, and the sense of number is also engaged to the perceptions and processing of visual, acoustic and linguistic stimuli, such as cognition of the number of sound pulses^31^ and categorization of multisyllabic utterances in the spoken language.^32^

### Cognitive informatics as a reverse engineering approach for rebuilding how the minds count

One may name that most impressive knowledge in the cognition study is the subconsciously processed powerful intelligent activity. Our daily thoughts are mostly carried out at far deep layers of the minds, as vast portions of the thoughts by humans are at non-conscious (subconscious) modes which are hardly accessible through conscious thoughts. As Lakoff and Nunez^19^ have summarized (in their book), the study on non-conscious “automated and instant” numeric comprehension is the basis for the origin of the mathematical concepts in human brain. Their hypothetical work is indicative that the most approaches to the arithmetic origins in cognitive capability are merely the reverse engineering attempts with mathematical frames targeting the cognition mechanisms naturally equipped in the human brain.

Cognitive informatics is one of such approaches to human cognition mechanisms backed by the mathematical models for reconstituting the neural processes as the mechanisms analogous to the informatic and computing models. In fact, cognitive informatics is relatively new discipline that studies the mechanisms for cognitive processes and the required internal information processes within the “natural intelligence,” chiefly, the brains and minds of humans.^33^ This field uses computing theories and informatics to solve the problems in neurosciences and cognitive psychology. Historically, in the related fields, a wide variety of ‘life functions’ have been identified,^34–39^ and it is considered that these ‘functions’ can be treated and described as the finite sets of fundamental cognitive processes in the brain.^40^

### Six-layered reference model for human brain

**Figure 1.**
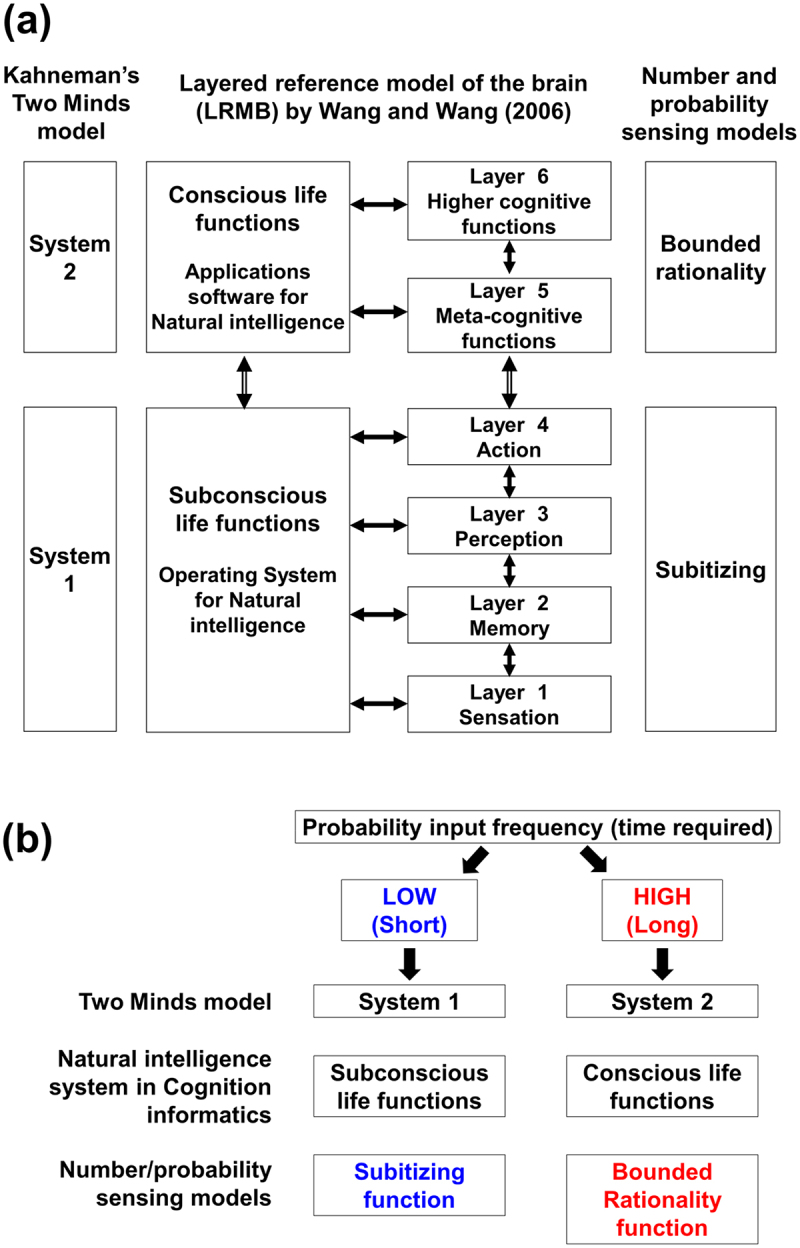
Comparison of the two minds, numeric and probability sensing, and the layered reference model for the brain (LRMB). (a) Based on similarity, system 1 (of TMT), basal four layers of cognitive processes in LRMB, and subitizing capability are aligned together (bottom) while system 2 (of TMT), top two layers of LRMB, and bounded rationality are aligned together (top). (b) Distinct roles for systems 1 and 2 in TMT, subconscious and conscious modes of intelligence in LRMB, and two distinct modes of probability sensing toward low and high input of *p*.

In order to describe a comprehensive set of mental processes and their inter-relationships, a hierarchical and integrated reference model of the brain has been proposed in cognitive informatics.^41^ Accordingly, a cognitive model of the brain and their relationships between the life functions have been depicted as the layered reference model for the brain (LRMB) consisting of six layers of cognitive processes, namely, the layers for (1) sensation, (2) memory, (3) perception, (4) action, (5) meta-cognition, and (6) higher cognition (Figure 1a). Wang and Wang^41^ described that total of 37 cognitive processes are being performed at each of six layers of the reference model.

In the above proposed LRMB framework, the non-conscious and the conscious processes are consisted of basal four layers (layers 1, 2, 3, and 4) and top two layers (layers 5 and 6), respectively. Within non-conscious layers, the sensational cognitive processes (in layer 1) cover the sensory inputs, namely, vision, audition, smell, tactility (of heat, pressure, weight, pain, and texture), and tastes (of saltiness, sweetness, bitterness, sourness, and pungency). The rest of non-conscious cognitive processes (in layers 2, 3 and 4) cover the principal life functions for memory, perception (self-consciousness, motivation, willingness, goal setting, emotions, sense of spatiality, and sense of motions), and action. Then, conscious meta-cognitive processes (in layer 5) cover functions for attention, concept establishment, abstraction, search, categorization, memorization, and knowledge representation. The highest cognitive processes (layer 6) are responsible for recognition, imagery, comprehension, learning, reasoning, deduction, induction, decision making, problem solving, explanation, analysis, synthesis, creation, analogy, planning, and quantification.

### Expanded subitizing and the probability sensing

Nowadays, the concept of subitizing has been expanded to the sense of probability (*p*). Téglás and his colleagues have revealed, in addition to simple numeric cognition, that preverbal human infants can reason about single-case *p* without relying on observed frequencies, by adapting their predictions to relevant dynamic parameters of the situation.^42–44^ Therefore, the sense of *p* can be dissected into the subitizing mode and the rational mode similarly to the numeric sense which could be dissected into the subitizing and the rational calculation involving distinct functions of the brain.

Decision-making for coping with the ever-changing environments are mental activities shaping and organizing human behavior. Most choice theories proposed by behavioral economists which are engaged in the study of decision-making, highly emphasize the “sense of *p*” as the key parameter as in the case of the expected value theory (EVT) which can be worked out from *p* of the value-gaining events. Therefore, the study on the sense of *p* rooted in the brain at the infancy is of great importance in the emerging field of behavioral economics.

Kahneman known for PT^5^ also proposed a dual system model called “Two Minds” or TMT focusing on two distinct modes of human cognitions for biases^3^ and morals,^4^ namely, System 1 typically responsible for intuition and indignation, and System 2 reflecting the rational mental functions.

Therefore, human behaviors can be viewed as the integration of outputs by System 1 (non-conscious automatic processes) and System 2 (conscious deliberate processes).^1,2^ System 1 reportedly activates a sequence of automatic actions while System 2 monitors System 1’s performance along with the existing plan and, at the same time, System 2 further activates future possible courses of actions.

Due to the close concepts between subitizing and intuition (System 1), and between the rational calculation and the slow thoughts (System 2), the authors highlight the similarities between “Two Minds” and two *p*-sensing modes in PT possibly based on subitizing capability and bounded rational thoughts. Therefore, in this study, Two Minds and two *p*-sensing modes are aligned along with the conscious and non-conscious functions of the brain (Figure 1a,b).

It is trivial to say that low-frequency events are less informative as predictors of the future, thus, the frequency or number of the events occurring has tight relationship with the future *p* required for forecasting the outcome. Therefore, as the information inputs (number of the event) increase, certainty (or *p*) also increases. In the below sections, how the “minds” sense *p* as the key information for decision-making is discussed.

## Novel mathematical models for probability sensing

### Theories for human actions based on the sense of *p*

PT explains the modes of human choice based on (1) the expected utility theory (EUT) derived from EVT and (2) the concept of weighting functions as discussed below.^45,46^ First of all, EVT, an early choice theory, calculates the level of wealth by directly relating *p* and outcomes. This theory makes the two options comparable and facilitates a smart choice. By viewing the value of each outcome as monetary rewards, *p* of each outcome can be expressed as the percentage choice of it occurring. By this way, EVT argues that each individual will select the choice as to maximize the return. The problem associated with EVT comes from the assumption that people act solely on calculation of the actual values of wealth (and the odds of receiving it). One may question if the motivation of individuals is always only monetary outcome or not. In reality, people want the monetary wealth for what it would be spent on, meaning that people will not only calculate a lottery based on the monetary gains, but also on how much they could be satisfied.^45^

Next theory to discuss is the EUT which handles the utility of wealth and the satisfaction derived from the wealth. Using EUT function, an individual’s utility of wealth can be plotted against actual wealth level. The visualized expected utility suggests that people are willing to achieve the best option available but they are somehow constrained by their own limitation and biases. It could be said that they are “boundedly rational”,^45^ and they do not seek to maximize the utility during the search for satisfaction.

As EUT is based on assertions about human behavior that are often missing in the real world, an alternative tool is required to model the human choice under risk and to predict the decision-making. Kahneman and Tversky’s PT is one such model challenging EVT and EUT, which incorporates the risk aversion or loss aversion common in individuals. PT made a major change to the earlier theories in a manner to weigh *p* of the event. While earlier theories simply weigh the value outcome against its actual *p* of occurrences, the PT introduced a “decision weight” that acts subjectively on that *p*. In case, *p* of a loss or gain may be 1% but it will not necessarily be assessed and acted upon as 1% in PT. As human always biases and limit the rationality, prediction of the human choice would be performed according to a weighting function. Since the point of modeling the human choice under conditions of risk is not to show what should happen, a theory should face what does happen in reality.^45^ Therefore, the decision weights used by PT are designed to mimic real human behavior.

### Weighting functions

PT’s decision weights on *p* defined as *d*(*p*), depicting *d* as an increasing function of actual *p*, and the higher the *p* the more likely that outcome will be the one decided upon.^45^ In this model, very low *p* receives overweighting with decision weights ensuring *d*(*p*) > *p*. The tendency to make overestimation at the low *p* range ensures that the rare events have a greater impact than they should. This implies that people worry more about very rare events and this appears to be an inherent characteristic of humans.^45^ While very rare *p* is over-weighted, this is compensated by a general trend to underweight the actual *p* of outcomes coming to pass. At high *p* range, the decision weight shows a greater divergence from actual *p*.

**Figure 2.**
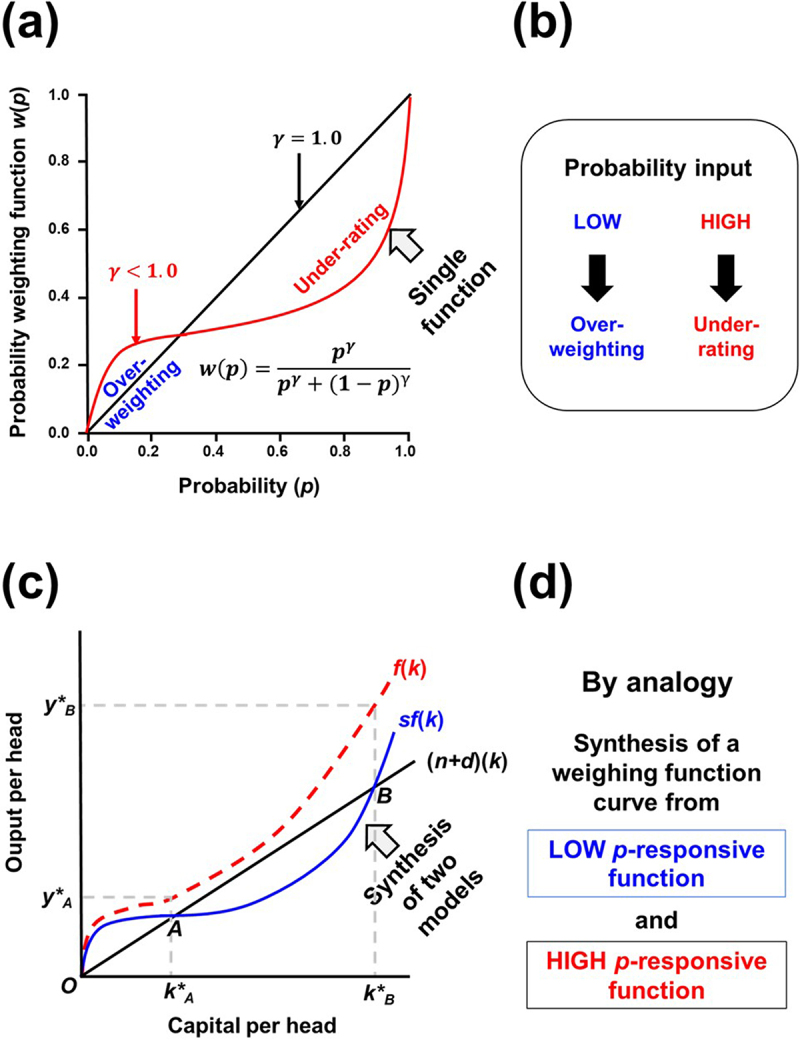
Examples of economic models with a single function and combined plural functions. **(a)** A typical curve with a weighting function in PT compared with 45° line linearly relating *p* and weighting. **(b)** Over-weighting of low *p* events and underweighting of high *p* events by a weighting function. **(c)** A production curve in macroeconomics explaining a world economy with no-growth (at point A) and high growth (at point B) as a synthesis of two distinct models. **(d)** Proposed synthesis of two separate functions to be applied as a pair of novel weighting functions.

As the results of biased weighting, the sum of all decision weights shrinks to a lower amount compared to the sum of all *p*. Whereas all *p* will sum to one, decision weight will have tendency to total less than this (*d*(*p*) + *d*(1-*p*) < 1). When plotted together with 45° line for decision weight straightly reflecting *p*, the overrated and underrated natures of a decision weighting function at very low *p* and high *p*, respectively, can be visualized (Figure 2a,b). These weights are then applied to the value of outcomes for gains or losses. The peculiarities of overweightness in the low *p* range and of underratedness in the high *p* range in a weighting function seem to have similarities with the System 1 and System 2 in TMT, respectively, as discussed later.

Proposal of PT by Kahneman and Tversky^5^ stimulated the researchers and as a consequence, a number of weighting functions have been proposed before Tversky and Kahneman^6^ proposed their own mathematical model, with a set of equations (eq. 1 and 2) each explaining the value and weighting bias, respectively. In fact, as discussed and emphasized by Nakamura,^47^ many types of equations all expressed as functions of given *p*, fulfilling the required characteristics as a weighting function (*i.e.*, over-rating and under-rating depending on the size of *p*) could be proposed.

(1) $$v\left(x\right)=\left\{\begin{array}{c} x^{\alpha },x\geq 0,\\ -\lambda {\left(-x\right)}^{\beta },x<0 \end{array}\right.$$

(2) $$w\left(p\right)=\frac{p^{\gamma }}{{\left(p^{\gamma }+{\left(1-p\right)}^{\gamma }\right)}^{\frac{1}{\gamma }}}$$

Note that a function (eq. 3) highly analogous to the eq. 2 has been proposed as a descriptive expression for EUT in 1978,^48^ which could be dated back even before the first proposal of PT in 1979.^5^

(3) $$w\left(p\right)=\frac{p^{\gamma }}{p^{\gamma }+{\left(1-p\right)}^{\gamma }}$$

In addition, eqs. 4–15 have been proposed and tested to date. While eq. 4 is consisted of discontinuous three distinct range of *p*, each of other formulae (eqs. 5–15) expresses the weighting nature as a single function.

(4) $$w\left(p\right)=\left\{\begin{array}{c} 0,p=0\\ \mathrm{\alpha p}+\beta ,0<p<1\\ 1,p=1 \end{array}\right.$$

Eq. 4 appeared in the early works by Bell,^49^ and followed by Cohen^50^ and Chateaune et al.^51^ and the following eqs, 5–8^52^ and eq. 9^53^ have been proposed in 1992, coinciding the proposal of eqs. 1 and 2 by Tversky and Kahneman.^6^

(5) $$w\left(p\right)=\frac{1-e^{-\mathrm{cp}}}{1-e^{-c}}$$

(6) $$w\left(p\right)=a+\mathrm{bp}+cp^{2}$$

(7) $$\log w\left(p\right)=a+b\;\log p+c\;\log p^{2}$$

(8) $$\log w\left(p\right)=a+be^{c\log p}$$

(9) $$w\left(p\right)=\frac{\alpha p_{i}^{\beta }}{p_{i}^{\beta }+\sum_{k-1}^{n}p_{i}^{\beta }}$$

Then, following eqs. 10–13^54^ and eq. 14^55^ have been proposed in the end of 20th century.

(10) $$w\left(p\right)=e^{-{\left(-\ln p\right)}^{\gamma }}$$

(11) $$w\left(p\right)=e^{-\beta {\left(-\ln p\right)}^{\gamma }}$$

(12) $$w\left(p\right)=p^{\beta }$$

(13) $$w\left(p\right)=\gamma e^{-\beta {\left(-\ln p\right)}^{\gamma }}$$

(14) $$w\left(p\right)=\frac{\delta p^{\gamma }}{\delta p^{\gamma }+{\left(1-p\right)}^{\gamma }}$$

The attempt to formulate novel weighting functions continues in this century as one of example by Rieger and Wang^56^ is shown below.

(15) $$w\left(p\right)=\frac{3-3b}{a^{2}-a+1}\left(p^{3}-\left(a+1\right)p^{2}+\mathrm{ap}\right)+p$$

Among the model functions proposed, equation 14 by Gonzalez and Wu^55^ provides very important information expressed as a factor *δ* designed to measure or weigh the mental attenuation named “attractiveness.” This is an attempt to isolate a component of mind function as a coefficient. The authors also found that the attractiveness *δ* could be useful and possibly be introduced into novel functions as discussed later.

### Toward homeomorphic two functions model

As listed above, weighting curves are likely expressed with single functions. Although these models nicely predict the behaviors of people, the authors question the legitimacy of such approaches by viewing these single functions as paramorphic models lacking the basis for two distinct modes of intelligence we have. As TMT suggests, the intuition-based decisions and the rationally made decisions could be attributed to distinct modes of human mind(s), and therefore, the overall weighting curve should be the synthesis of two distinct functions homeomorphically representing the characteristics of two independent minds.

In economics, two distinct mechanisms behind a single curve in a graph is often studied. For an instance, a case of a production function *f*(*k*) and a derived saving line *sf*(*k*) depicting the relationships between the productive output per head (*y*) and the capital input per head (*k*) such as one example appeared in a macroeconomics text book authored by Dornbusch et al.^57^ can be named, in which two kinds of investment opportunities could be found, namely, (i) those with diminishing marginal product as in the neoclassical growth model (Figure 2c, between O and A), followed by (ii) those with constant marginal product as often found in the endogenous growth model (Figure 2c, at point B). In this case, the production curve begins with a highly curved section and ends up with an upward-sloping line. Therefore, at low capital input, the line of investment required to maintain the level of capital, (*n+d*)*k*, strikes the derived saving line in the neoclassical region (at point A), then followed by a no-growth steady state. On the other hand, under high capital input (passing the point B), the saving line goes above the capital reequipment line and goes on growing.

By analogy to this example from data handling in macroeconomics, the weighting function in PT could be dissected into a pair of functions, one responsible for low *p* events (subitizing) and the other responsible for high *p* events (bounded rationality).

## Canopy leaf-inspired paired Hill-type functions for probability weighting and derived paired cognition automata

### Learning from biological processes

In green plants, light energy is processed through the layers of leaves, just like information processing through the layers of cognitive processes in the human brain. By observing living trees, a classical botanist Boysen-Jensen^58^ noticed that the leaves positioned at the top of trees (sunny leaves) and the leaves located deep inside the canopy structure (shade leaves) show different capacities for light. Sunny leaves show higher photosynthetic capacity under intensive light and the photosynthesis in the shade leaves is soon saturated under relatively low light intensity. This difference in photosynthetic capacity in two-types of leaves has reason.

**Figure 3.**
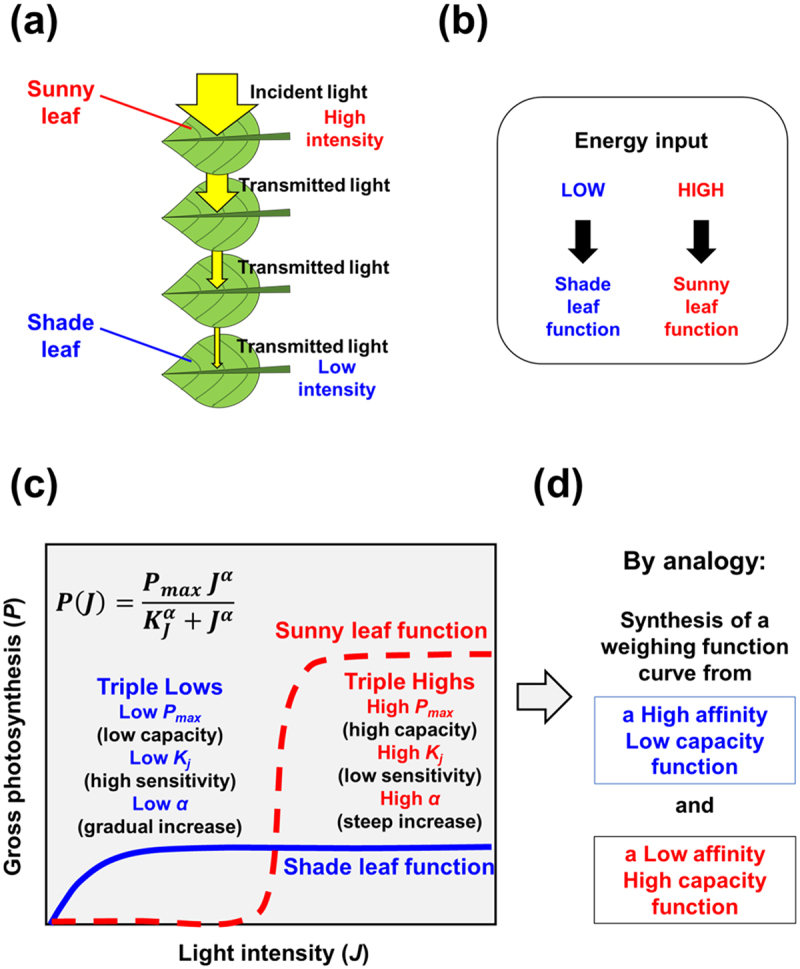
Learning from the photosynthesis through the layered leaves. (a) Light energy processing through leaf layers. (b) Possible distinct functions in different leaf layers. (c) Naturally found paired functions for photosynthesis in shade and sunny leaves sharing the identical mathematical frame. (d) Proposed synthesis of a weighting curves from a pair of distinct functions.

Light is filtered through each leaf layer and the transmittance light passes through at a specific rate (Figure 3a,b). Through recurrent light harvesting by the layers of leaves, light energy is effectively collected and utilized for photosynthesis. As a consequence, the leaves at the top and bottom positions have distinct roles (Figure 3c). In the top sunny leaves, high capacity for intense light is required while giving up the sensitivity (affinity) to the low intensity light range. In the bottom shade leaves, effective capturing of low intensity light with high affinity is achieved while giving up the capacity for intensive light.

Previously, Kawano and his colleagues have proposed an equation (eq. 16, one of Kawano’s photosynthetic equations) for photosynthetic gross production (*P*) as a function of light intensity (*J*),^59,60^ by converting Platt-Jasby equation which has similar structure to Michaelis-Menten-type equation applicable to the study of photosynthesis under light emitting diodes^61^ into a Hill-type equation by introducing an exponent α.^62,63^

(16) $$P\left(J\right)=\frac{P_{\max}J^{\alpha }}{K_{j}^{\alpha }+J^{\alpha }}$$

Unlike conventional mathematical models for photosynthesis, this equation (equation 16) can be applied to, and reproduce the light response curves of photosynthesis in both the shade and sunny leaves which are highly differed in sensitivity (expressed as a constant for light requirement, *K*_*j*_ which is equivalent to Michaelis constant), capacity (expressed as *P*_*max*_), and steepness of the slope (expressed with an exponent *α* known as Hill’s coefficient). This suggests that plant leaf canopy is consisted of the sunny-positioned leaves with three high factors (high *P*_*max*_, *K*_*j*_, and *α*), and the shade-positioned leaves with three low factors (low *P*_*max*_, *K*_*j*_, and *α*) (Figure 3c). Therefore, overall photosynthesis in a whole tree must be the synthesis of different functions.

In nature, there are plenty of examples for showing the ‘trade-off’ between the sensitivity (affinity, specificity, or accuracy) and capacity (or speed), *i.e*., a system with low affinity (high *K*_*M*_) plus high capacity versus high affinity (low *K*_*M*_) plus low capacity as found in the behaviors of transporters in plants,^64,65^ planktons,^66^ and human brains^67^ and functions of transcription factors.^68^ By analogy, we propose here that a *p*-weighting curve could be composed of a pair of functions differed in affinity and capacity, sharing the structure of Hill’s equation. It is assumed that the paired Hill-type equations, one with 3 lows (low *S*_*max*_, *K*_*S*_, and *α*_*S*_) and the other with 3 highs (high *R*_*max*_, *K*_*R*_, and *α*_*R*_) correspond to the subitizing capability (eq. 17) and the rational mental activity (eq. 18), respectively. Note that the symbols *S* and *R* stand for subitizing and bounded rationality, respectively.

(17) $$F_{S}\left(p\right)=\frac{S_{\max}p^{\alpha_{S}}}{K_{S}^{\alpha_{S}}+p^{\alpha_{S}}}$$

(18) $$F_{R}\left(p\right)=\frac{R_{\max}p^{\alpha_{R}}}{K_{R}^{\alpha_{R}}+p^{\alpha_{R}}}$$

Equation 17 with low constant (*K*_*S*_) depicts the behavior at low range of *p* just above *p*  = 0. By considering *p*  = 0 as a threshold for subitizing action, *K*_*S*_ should be set close to *p*  = 0. Equation 18 with high constant (*K*_*R*_) depicts the actions at higher range of *p* beyond the threshold point (*T*). Since rationality must be activated only above *T*, *K*_*R*_ should be set close to, but above *p* = *T*.

### Cognitive processes as automata

**Figure 4.**
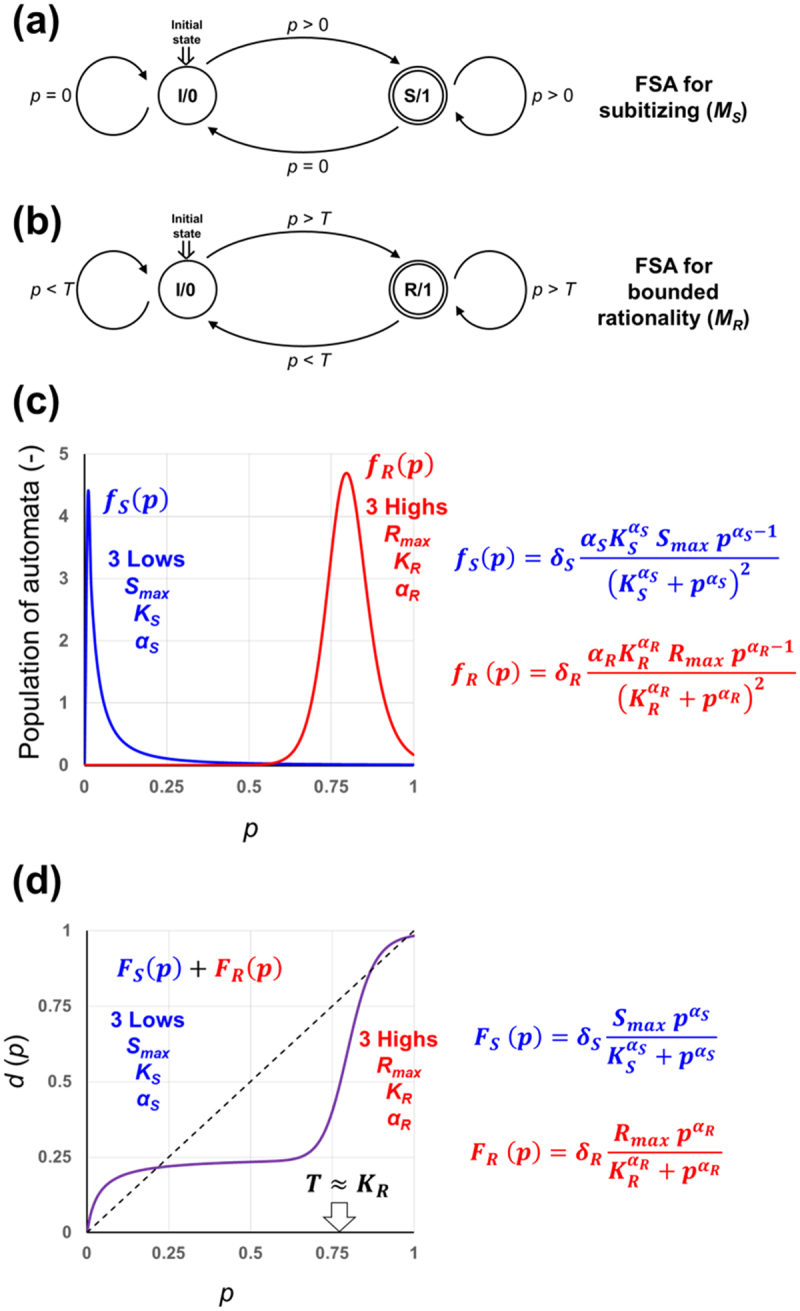
Two finite state automata (FSA) models corresponding to subitizing (S) and bounded rationality (R). (a) FSA *M*_*S*_ = (*Q*_*S*_, *Σ*_*S*_, *Δ*_*S*_, *q*_*S*0_, *F*_*S*_), where a finite set of states is consisted of the initial (I) and the subitizing (S) states, thus, *Q*_*S*_, = {I, S}; a finite set of inputs are *p = *0 *and p >* 0, *thus, Σ*_*S*_ = {*p = *0, *p >* 0}; the state transition function *Δ*_*S*_ involves the state and input, thus, *Δ*_*S*_ (I, *p = *0*) = *I, *Δ*_*S*_ (I, *p >* 0*) = S, Δ*_*S*_ (S, *p = *0*) = *I, *Δ*_*S*_ (S, *p >* 0*) = S; q*_*S*0_*=* I, *F*_*S*_ = S. (b) FSA *M*_*R*_ = (*Q*_*R*_, *Σ*_*R*_, *Δ*_*R*_, *q*_*R*0_, *F*_*R*_), where *Q*_*R*_, = {I, R} whereas I and R stand for the initial state and the bounded rationality state, respectively; *Σ*_*R*_ = {*p < T*, *p > T*} whereas *T* is the threshed *p* beyond which transition from I to R is activated; *Δ*_*R*_ (I, *p < T*) = I, *Δ*_*R*_ (I, *p > T*) = R, *Δ*_*R*_ (R, *p < T*) = I, *Δ*_*R*_ (R, *p > T*) = R; *q*_*R*0_*= I, F*_*R*_ = R. (c) possible distribution of automata (*M*_*S*_ and *M*_*R*_) at work explaining the *p*- weighting. (d) Synthesis of two functions to perform *p*-weighting.

Prior to further discussion on the behavioral sensitivity to *p*, which is to be expressed as weighting functions with equations 17 and 18, some possible modeling of the subitizing and the bounded rationality responsive to *p* input by viewing them as automata are attempted here (Figure 4). By assuming that a neuronal unit within the brain can be considered as an abstract sequential machine, the signal processing inside the brain can be collectively expressed as automaton-like decision-making processes by each unit. Therefore, the functioning units relaying signals upon perception of stimuli must be interpreted either as Mealy or Moore machines.^22^

Since a Moore machine can be converted to a finite state automaton (FSA), the systems of interest are now described as a pair of Moore-type FSAs sensing *p via* subitizing mechanism (*M*_*S*_) and bounded rationality (*M*_*R*_). By definition, an FSA (*M*) can be expressed by a quintuple, *M* = (*Q*, *Σ*, *Δ*, *q*_0_, *F*): where (1) *Q* represents a finite set of states; (2) *Σ* represents a finite set of inputs; (3) *Δ* represents the state transition function determining the next state *r* (∈Q) according to the combination of the present state *q* (∈Q) and the input *a* (∈Σ), providing that *Δ*(*q*, *a*) = *r* ; (4) *q*_0_ (∈Q) represents the start state, that is, the resting (initial) state before any input is applied; and (5) *F* is a set of final states of *Q* (*i.e. F*⊆Q).

In Figure 4(a,b) the states allowed in these FSAs are represented by circles, and the transitions are represented by the arrows. The initial state in each model is shown by the double arrow and the final state is shown with the double circle. Inputs (represented by *p*) inter-connect the initial and final states.

A brain is an organ with collective intelligence in which the data processing must be collectively performed by a number of sensing and signaling neural units with varying thresholds. Therefore, *p*-driven decision mechanisms should not be modeled with a single automaton or a pair or set of two or several single automata, but instead there should be a set of mass of automata with varying sensitivity which should be naturally showing broad distribution patterns with tailing which are different from the normal bell-shaped distribution (Figure 4c), as discussed in the prior reports.^22,25,69^ These distribution patterns (Figure 4c; equations 19 and 20) are derived by differentiation of Hill-type equations proposed for subitizing and bounded rationality (eqs. 17 and 18). *Vice versa*, by integrating the distribution of automata at work as in eqs. 19 and 20, the paired functions (eqs. 17 and 18) for *p*-weighting curve can be obtained (Figure 4d).

(19) $$f_{S}\left(p\right)=\frac{\alpha_{S}\;S_{\max}\;K_{S}^{\alpha_{S}}\;p^{\alpha_{S}-1}}{{\left(K_{S}^{\alpha_{S}}+p^{\alpha_{S}}\right)}^{2}}$$

(20) $$f_{R}\left(p\right)=\frac{\alpha_{R}\;R_{\max}\;K_{R}^{\alpha_{R}}\;p^{\alpha_{R}-1}}{{\left(K_{R}^{\alpha_{R}}+p^{\alpha_{R}}\right)}^{2}}$$

Here, by introducing the factor similar to the attractiveness factor *δ* of Gonzalez and Wu^55^ determining the ratio of contribution by each mode (*δ*_*S*_ *+ δ*_*R*_ = 1), eqs. 17–20 can be further modified into eqs. 21–24 when required. In fact, the graphs in Figure 4(c,d) are attenuated by introduction of *δ*_*S*_ (0.25) and *δ*_*R*_ (0.75).

(21) $$f_{S}\left(p\right)=\delta_{S}\frac{\alpha_{S}\;S_{\max}\;K_{S}^{\alpha_{S}}\;p^{\alpha_{S}-1}}{{\left(K_{S}^{\alpha_{S}}+p^{\alpha_{S}}\right)}^{2}}$$

(22) $$f_{R}\left(p\right)=\delta_{R}\frac{\alpha_{R}\;R_{\max}\;K_{R}^{\alpha_{R}}\;p^{\alpha_{R}-1}}{{\left(K_{R}^{\alpha_{R}}+p^{\alpha_{R}}\right)}^{2}}$$

(23) $$F_{S}\left(p\right)=\delta_{S}\frac{S_{\max}\;p^{\alpha_{S}}}{K_{S}^{\alpha_{S}}+p^{\alpha_{S}}}$$

(24) $$F_{R}\left(p\right)=\delta_{R}\frac{R_{\max}\;p^{\alpha_{R}}}{K_{R}^{\alpha_{R}}+p^{\alpha_{R}}}$$

## Decisions after decisions

### Preference reversals on the course of collecting information

It is natural to assume that people keep thinking on the same topic and sometimes the final decision becomes different from the earlier one, especially in the case that the incomplete pieces of information were initially provided, or different types of information collectively complementing each other to build the entire image on the same topic of interest are provided at different timings. Therefore, people recurrently think each time when new piece of information is provided. By asserting that uncertainty decreases as the information is enriched with additional news, the overestimation of values made at the earlier timing under the limited access to the information should be corrected in stages.

TMT argues that System 1 is responsible for the fast/immediate decisions and System 2 is responsible for the long-term decision. It is reported that the decisions rapidly made through System 1 can be canceled through the long rational thoughts by System 2.^2^ In behavioral economics, such reversals of preference or decisions are hot topics to be discussed, especially in PT.^70^ Some cases could be naturally explained as below.

Cumulative prospect theory (CPT)^6^ built on the earlier PT model is designed to target the source of its flaws found in the earlier PT models, which is the lack of distinction made between ‘gains’ and ‘losses’ in the decision weighting function. Original PT^5^ was designed to assign each *p* value a unique decision weight and to apply this equally to both the gains and losses. Therefore, even if people acknowledge that the gains and losses are valued differently, the original PT ignores the cases deserving different *p*-decision weights for the gains or losses.

Let the decision weighting for positive and negative gains (gains and losses) upon inputs of *p* to be designated as *d*^*+*^(*p*) and *d*^*−*^(*p*), respectively. While in the original PT, gains and losses are equally assessed as *d*^*−*^(*p*) = *d*^*+*^(*p*), in CPT, gains and losses are differently scored as *d*^*−*^(*p*) = 1− *d*^*+*^(1−p). The changes made in CPT resolve the problems around decision weightings that hindered the original model, replacing the set weights for each *p* value with a cumulative weighting function that incorporates gains and losses.

Now we reach the most serious problem with original PT (and EUT) which is remained unsolved since the amended model (CPT) also hardly predict the preference reversals. In case of buying a gamble option, one may agree to pay 100 USD for owning a gamble, in turn, when asked for selling it, one may feel like to release the gamble at higher price. This example is a typical case of preference reversals.^70^ As similarly to well known ‘sunk cost effect’ or ‘Concorde effect’ in decision-making, personal history with preliminary costs or event experienced, largely matters. Assuming that a preference reversal occurs as a secondary decision, these cases could be also viewed that preferences or preliminary decisions could be reversed or canceled as a consequence of recurrent thoughts made after collection of information through further experiences.

Again, let’s think of a situation to sell a gamble. We can sell it only after owing one, thus this is possible only after the first choice and action to buy and own. Note that the secondary choice can be made after the evaluation of the information accompanying and/or following the first decision. In case that the first decision was forced to make with minimal information provided, the second choice could be made after additional collection of information (experience). It is also likely to happen that recurrent thoughts repeatedly confirm or strengthen the initial decision or the decision with one direction is reversed in the final decision. We need a homeomorphic model to explain the nature behind these decisions.

### Habituation as a special case of preference reversal

The second case to discuss is the habituation. Research has shown that animal and humans habituate on a variety of behavioral and physiological responses to repeated presentations of stimuli, such as food cues.^71^ In animal studies, analysis of habituation is widely used to characterize cognitive phenotypes and such study now covers even fishes.^72^

In case of receiving an alert or sign of danger, it is safe to make an immediate action to avoid or to evade from the source of danger without conducting detailed analysis of the quality of information. This immediate decision required for survival could be smoothly performed through subitizing capability or by System 1.

**Figure 5.**
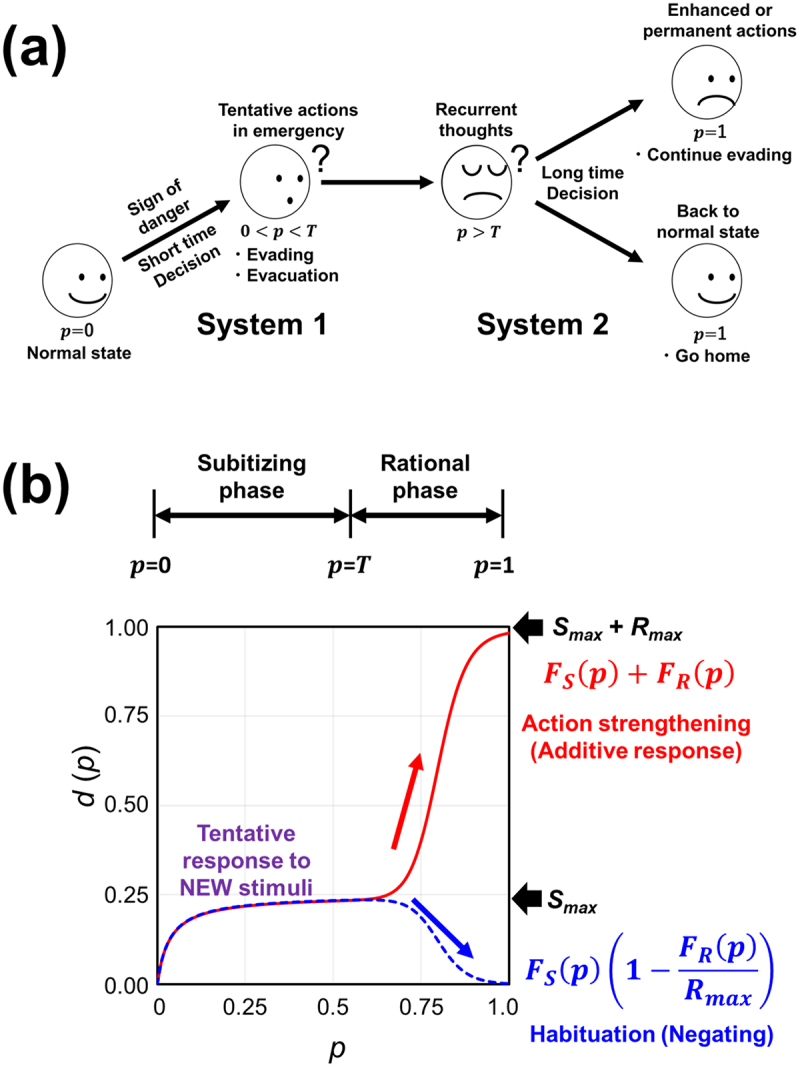
Models for recurrent decision making along with enrichment of information leading to increment of *p*. (a) Flow of repeated decision making upon facing *p* of dangers ending with diversified final decision in either positive or negative manner. (b) A model for explaining the reversal or enhancement of initial decision.

The authors see that the processes to cause habituation and preference reversal may have similarity. If the habituation or preference reversal occurs, the rational mode responsible for the analyses of frequent information inputs in stages must take part. Assuming that the tendency to switch (reverse) the preference or decision can be expressed as a function of *p*, overall decision model with a pair of distinct functions is proposed here, of which, one is responsive to rare (very low frequency) event and the other is responsive to the events with higher frequency (Figure 5). Following equations can be bifurcating examples of mathematical models for describing the confirmation or the reversal of earlier decisions (eqs. 25, 26).

(25) $$d\left(p\right)=F_{S}\left(p\right)+F_{R}\left(p\right)$$

(26) $$d\left(p\right)=F_{S}\left(p\right)\left(1-\frac{F_{R}\left(p\right)}{R_{\max}}\right)$$

Models described in Figure 5 explain how decisions are recurrently made along with enrichment of information often resulting in habituation to the repeatedly received sign of dangers. As shown above, it can be said that this is a model in which System 1 and System 2, or subitizing and rational thoughts, work cooperatively but in tandem. It remains interesting to know how the bifurcation into either of the modes with equation 25 or equation 26 is controlled, but at present, we can say that we have prepared a pair of mathematical models simply expressing two distinct actions to be taken.

## Plants’ memory-oriented behavioral reversal

As we initiated discussion of mathematical models in the upper half of this paper by introducing a metaphoric plant photosynthetic model, here again, we wish to return to the discussion of plants’ behavior. In fact, habituation is the phenomenon sometimes observed in plants too. In order to perform habituation, certain level of memory is required for assessing the types of incoming signals as new or known. If plants have memory or not, is one of hot topics discussed today. We have previously discussed that short-term memory can be stored and processed via cumulative electro-physiological signals in automaton-like manner in *Dionaea muscipula*,^22^ and the presence of long-term memory in *Mimosa pudica* was demonstrated by Gagliano et al.^73^ In fact, well-planned experiments with *Mimosa pudica* by Gagliano et al.^73^ testifying the plants’ ability to distinguish the different types of mechanical stimuli given after long intervals, thus, proving the long-term memory in plants, were inspired by a classical work performed two centuries ago, as described in a series of books authored by Mancuso and Viola^74^ and Mancuso.^75^ Mancuso’s books cited a classical book authored by De Lamarck and De Candle^76^ which is preserved as a part of Sorbonne Collection at Centre Franco-Japonais d’Histoire des Sciences in Kitakyushu, Japan,^77,78^ which Mancuso, Baluška, and Bouteau accessed and read as they gathered in Kitakyushu in 2006.

In the 19th century book mentioned above, a pioneering work by René Desfontaines (1750–1833) was collected. Accordingly, Desfontaines engineered an unusual experiment monitoring the behavior of *Mimosa* plants during a long tour by carriage on the paved streets of Paris, thus, exposing plants to continuous vibrations inevitably causing closure of leaves at once but followed by observation that “the plants are getting used to it.” Yet the implications of Desfontaines’s test were clear even back then, and they pointed decidedly to an adaptive behavior in plants resulting from the storage of information.^75^

**Figure 6.**
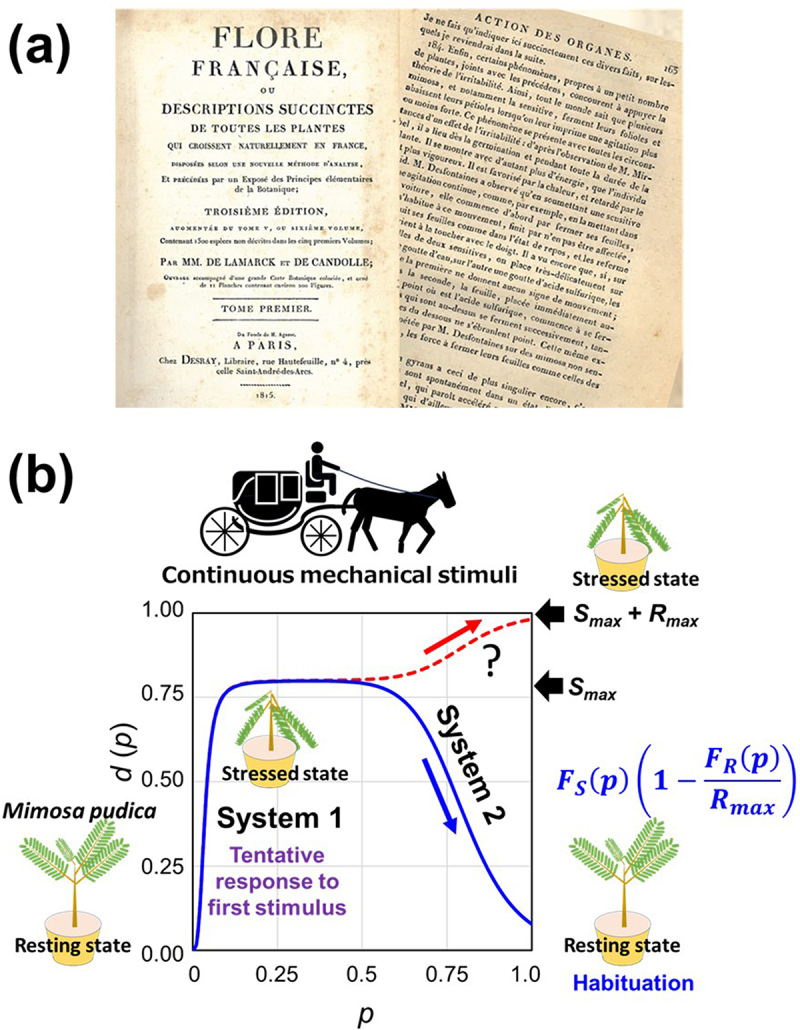
Habituation to the repeated stimuli in *Mimosa pudica*. (a) Front page and the page reporting the work by René Desfontaines, in De Lamark and De Candolle^76^ Flore Françoise, a collection at Centre Franco-Japonais d’Histoire de science. (b) Historical demonstration of habituation in *Mimosa pudica* transported in the carriage running on the stone-paved roads in Paris being exposed to repeated mechanical stimuli.

This classical episode by Desfontaines^76^ and its redemonstration by Gagliano et al.^73^ not only teach us the presence of memory in plants but also the behavior of plants to be described as habituation which is comparable with the preference reversal found in human behaviors possibly consisted of two phases of decision-making procedures equivalent to System 1 and System 2 (Figure 6). Then, what is the likely conclusion from the comparison of the human behavior in Figure 5 and the plant behavior in Figure 6, both sharing the habituation to continuous input of information increasing the size of *p* in stages? If we can consider that habituation is based on the mechanism involving Two Minds, the fact that plants show habituation as in the case of *Mimosa* plants upon exposure to the repeated mechanical stresses, we dare to say that plants also have Two Minds or at least the functions or capability equivalent to Two Minds of humans.

Historically, Aristotle denoted that plants have vegetative souls which are seriously impaired rational and perceptual capacities, thus, way lower or primitive compared to the animal souls.^74,79^ Here, we do not argue that which one (vegetative soul or animal soul) to be higher or lower, but simply accept the fact that both plants and animals behave similarly upon continuous input of information.

## Expanded concepts and evolutionary origins of two minds

Here, we wish to propose that the expanded concepts of System 1 and System 2 possibly shared in animals and plants could be rooted from the common nature preserved among a wide spectrum of organisms from prokaryotic unicellular species to the higher complex organisms.

As we have highlighted the common behaviors of plants and animals through the above conceptual frameworks, the possible origin of the nature of Two Minds (Systems 1 and 2) emerged out along with evolution must be discussed. Here, a number of questions come out of discussion. (1) Did System 1 arise with the beginning of life and preserved in all organisms? (2) What is the neurobiological basis for System 1? (3) After confirming the presence of System 2 in both plants and animals, then, does System 2 equally reside in organisms with multicellularity but with sessile nature as in the case of plant with sessile nature? (4) Is System 2 omnipresent in all multi-cellular organisms, thus, including the member of fungal Kingdom too? (5) Or in fact, is System 2 omnipresent in all eukaryotic organisms including unicellular protist? (6) Assuming that System 2 is omnipresent in all organisms with multicellularity and mobile nature, how is it backed by neurobiological structure and process?

**Figure 7.**
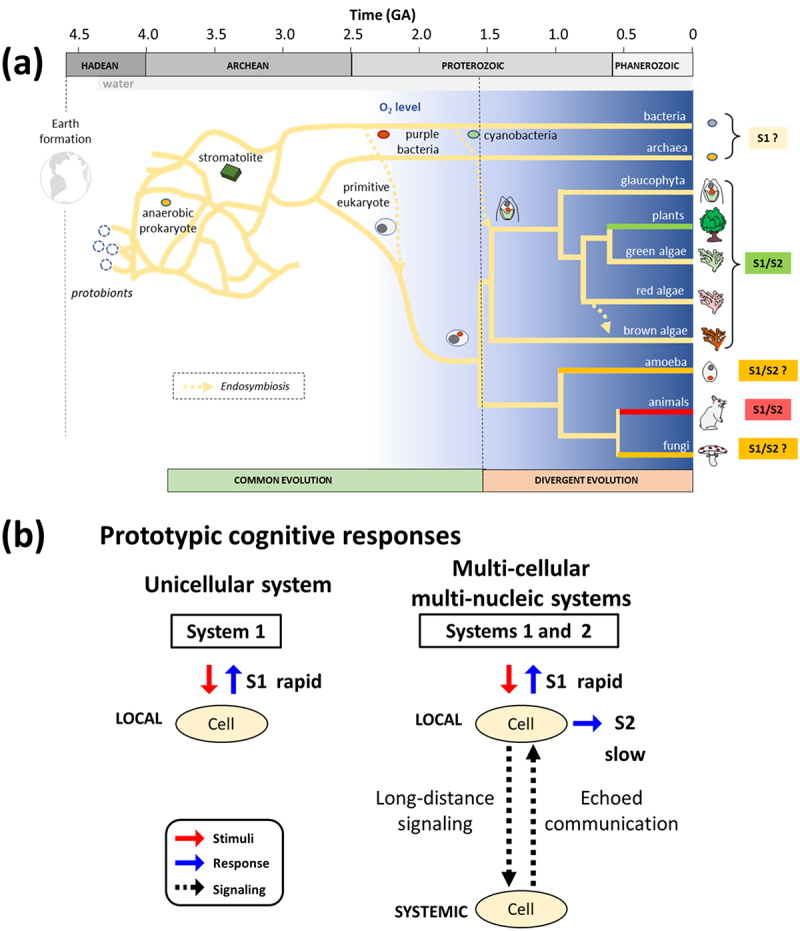
Possible emergence of two minds in the course of evolution. (a) Two distinct phases of evolution (common phase and divergent phase). Macroevolution through intracellular symbiosis is illustrated (arrows with broken lines), according to the intracellular symbiosis theory, showing that organelles such as chloroplasts have repeatedly migrated among the organisms, originally initiated as incorporation of a small prokaryotic cell into a large prokaryotic cell occurred. (b) Simplified models for explaining the emergence of two minds in multi-cellular organisms.

It has been elucidated that life showed divergence into plants and animals *ca* 1.6 gigaannum ago（GA, or billion years ago; Figure 7a, image modified from Boutau et al.^80^). Based on our finding that plants showed the modes of actions similar to Two Minds, we view here that at least System 1 in both human (and animals) and plants could be rooted from the earlier stage of evolution prior to the plant-animal divergence (Figure 7b).

**Figure 8.**
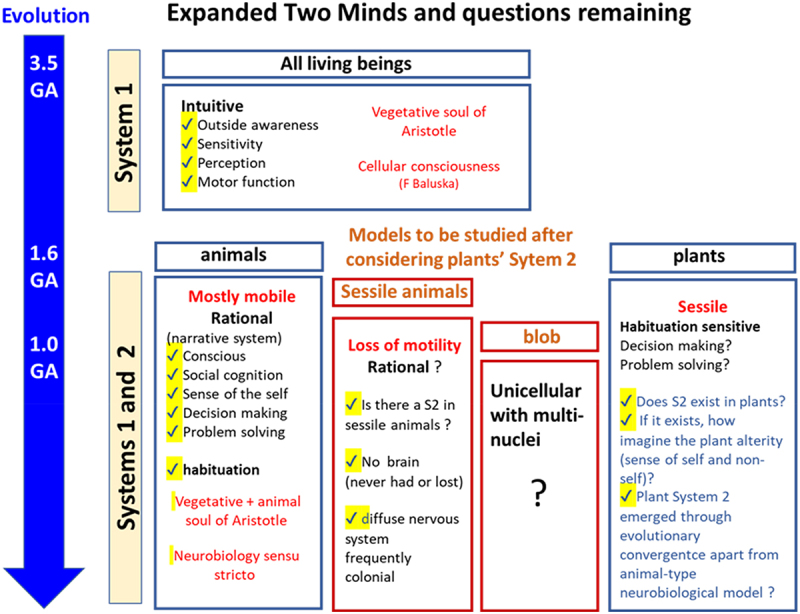
Model organisms to be studied and the discussions remaining.

Arthur Reber, František Baluška and colleagues have proposed that every cellular organism from the small-sized unicellular organisms to the large-sized multi-cellular organisms might have developed a mode of primitive state of cellular reactions to be considered as cellular consciousness involved in cognitive adaptations to the ever-changing environmental stimuli largely contributing to survival of the organisms^81–86^. In the following discussion, for the sake of simplification, let us temporally rephrase Reber-Baluška’s cellular consciousness shared among all cellular organisms as System 1 (Figure 8, upper).

Having accepted that Two Minds can be found in both vascular plants and metazoans, which diverged *ca*. 1.6 GA in evolutionary time, it is now natural to assume that there could be other organisms with Two Minds that diverged from these groups, such as fungi, which are thought to have diverged from animals in the Phanerozoic eon (Figure 8). Unicellular eukaryotes such as *Physarum polycephalum*, an acellular slime mold popularly known as the blob, belonging to the Amoebozoa, which diverged from animals and fungi around 1 GA, could also be the candidate organisms with extended Two Minds behaviors (Figure 8, lower), as the blob is a well-known intelligent member of the Amoebozoa, capable of cognitive behavior and natural computation to solve mathematical problems,^28,87^ this organism must therefore be a good model for investigating the presence of intelligence in Two Minds forms. Studying organisms such as non-plant sessile organisms, especially sessile animals such as coral species that belong to the metazoans but have a sessile life cycle, would be of interest to understand possible differences in the Two Minds systems in plants and animals (Figure 8, lower). For an instance, a recent study revealed that the common Caribbean coral *Porite astreoides* judges when and where to settle down by listening to the surrounding environmental sounds.^88^

Biologists today explain the biological phenomena using the terms in chemical biology and biophysics often focusing on the (1) reactions altering or modifying the materials composing the organisms and (2) the flow of energy, since all biological systems obey the laws of physics and chemistry. In addition to materials and energy, the third factors shaping the biological phenomena is “information”.^59^ The modes of cognition and decision-making being discussed here totally function on the basis of perception and processing of information.

N. Wallbridge and C. Plumer have proposed a theory with a simulative work performed together with T. Kawano^59^ that the physical distance required for combined exchanges of electrical and chemical signals within the multi-cellular organism strongly influence the physiology of multi-cellular structure. Furthermore, the theory explains that the body size limitation in each organism and the intrinsic growth rate reflecting the cell’s capability to divide are largely limited by the velocity of the propagation of information within the entire body of an organism with multi-cellular nature. Assuming that the rate of cell division (leading to an increase in total size of an organism) has tight relationship with the number of communicating and/or signaling events required for collective local growth under well-organized systemic controls. Then, the relationship between the growth rate and the maximal size of an organism could be subjected to the trade-off regulated by the velocity of the flow of information throughout the whole organism.

To orchestrate the cellular events which have tendency to increase the number of tasks per unit of time (frequency), which is proportional to the rate of growth (cell division rate) in all the cells within the individual organism, either of the growth rate or the body size of the organisms must be restricted. Upon perception of external stimuli at one cell or at a tip of an organ, a local response could be immediately induced but coordinated responses integrating the information from the other portion of the organism must be taken with certain time-lag, and the extent of such time-lag must be determined by the velocity of cellular communication, and the distance of the communicating path reflecting the physical size of organism. Now, we would like to bring the analogies (i) between the local immediate cellular responses and System 1 and also (ii) between the systemically echoed response with time-lags and System 2.

Taken together, it could be deduced that unicellular organism or very small multi-cellular organism shows only the local immediate responses without systemic echoing of the signals with time-lags, thus, only the response equivalent to System 1 may exist in unicellular organisms. In contrast, in large-sized plants and animals with multi-cellular structure, the mode, quality, and timing of local and systemic response could show divergence depending on the size of organisms, velocity of signal propagation per cell, and the type of intelligence employed, i.e. with centralized animal nerve and brain system or decentralized plant-type collective data processing involving root apex.^89–92^ In the Cellular Basis of Consciousness (CBC) theory, only the cells have the primary version of consciousness^82,85,86^ whereas organisms based on multiple cells assemble the corporate version of consciousness and life.^93^ Multicellular organisms do not have direct access to the primary cellular consciousness and perceive it only indirectly, as subconscious feelings-based intuitions of the System 1.

## Conclusion and perspectives

This paper focused on the similarities among non-consciously processed rapid decisions through System 1, overweighing of *p*, and the subitizing toward *p*, followed by slow decision made by consciously processed System 2, underrating of *p*, and the rational analysis of *p*. While most of reported *p*-weighting models for PT employed single functions, this paper proposes a pair of Hill-type functions for weighting of *p* reflecting the collective behaviors of two types of automata corresponding to subitizing and rationality. The mathematical structure for the paired functions is inspired from the assorted photo-energy processing in the layered leaves of living plants.

Today, the collaborating teams of the authors are interested in the search for the signs of the presence of Two Minds in a wide variety of biological systems including lower animals, microbials and plants. Furthermore, we are hoping that this decision-making concept could be expanded from the models with individuals to the models with the groups of organisms and/or humans. Then, social behaviors of animals in the ecosystem and decision-making by groups, teams, units, and entities of people with social, political, and commercial aspects could be possibly covered.

If any additional evidence of recurrent thoughts during decision-making leading to preference enhancement or preference reversal (as in the case of habituation) could be found in the above-mentioned models, this view could be largely expanded and strengthened.
